# The effect of continuing versus withholding angiotensin-converting enzyme inhibitors/angiotensin II receptor blockers on mortality and major adverse cardiovascular events in hypertensive patients undergoing elective non-cardiac surgery: study protocol for a multi-centric open-label randomised controlled trial

**DOI:** 10.1186/s13063-022-06616-y

**Published:** 2022-08-17

**Authors:** Satyajeet Misra, Satyen Parida, Raj Sahajanandan, Bikram Kishore Behera, Muthapillai Senthilnathan, Ramamani Mariappan, Tony Thomson Chandy, For the ACE investigators

**Affiliations:** 1 AIIMS Bhubaneswar, Bhubaneswar, Odisha 751019 India; 2 JIPMER Puducherry, Puducherry, India; 3 CMC Vellore, Vellore, India

**Keywords:** ACE inhibitors, ARB, Acute kidney injury, Angioedema, Complications, Mortality, Outcomes

## Abstract

**Background:**

Angiotensin-converting enzyme inhibitors (ACEIs)/angiotensin receptor blockers (ARBs) are commonly prescribed to patients with hypertension. These drugs are cardioprotective in addition to their blood pressure-lowering effects. However, it is debatable whether hypertensive patients who present for non-cardiac surgery should continue or discontinue these drugs preoperatively. Continuing the drugs entails the risk of perioperative refractory hypotension and/or angioneurotic oedema, while discontinuing the drugs entails the risk of rebound hypertension and myocardial ischaemia. The aim of this study is to evaluate the effect of continuation vs withholding of ACEIs/ARBs on mortality and other major outcomes in hypertensive patients undergoing elective non-cardiac surgery.

**Methods:**

The continuing vs withholding of ACEIs/ARBs in patients undergoing non-cardiac surgery is a prospective, multi-centric, open-label randomised controlled trial. Two thousand one hundred hypertensive patients receiving ACEIs/ARBs and planned for elective non-cardiac surgery will be enrolled. They will be randomised to either continue the ACEIs/ARBs including on the day of surgery (group A) or to withhold it 24–36 h before surgery (group B). The primary endpoint will be the difference in the composite outcome of all-cause in-hospital/30-day mortality and major adverse cardiovascular and non-cardiovascular events. Secondary endpoints will be to evaluate the differences in perioperative hypotension, angioneurotic oedema, myocardial injury, ICU and hospital stay. The impact of the continuation vs withholding of the ACEIs/ARBs on the incidence of case cancellation will also be studied.

**Discussion:**

The results of this trial should provide sufficient evidence on whether to continue or withhold ACEIs/ARBs before major non-cardiac surgery.

**Trial registration:**

Clinical Trials Registry of India CTRI/2021/01/030199. Registered on 4 January 2021

**Supplementary Information:**

The online version contains supplementary material available at 10.1186/s13063-022-06616-y.

## Background

Annually, more than 280 million people undergo non-cardiac surgery [1]. The mortality rate following non-cardiac surgery is 0.3% [2]. This risk is higher in patients with ischaemic heart disease, diabetes and hypertension. The perioperative period is the most vulnerable period to develop cardiac or vascular complications which are seen in 25% of patients undergoing major abdominal, vascular and orthopaedic surgeries [2]. More than 1/3 of the patients coming for non-cardiac surgery who present with hypertension may be receiving angiotensin-converting enzyme inhibitors (ACEIs)/angiotensin II receptor blockers (ARBs) which are commonly prescribed as first-line anti-hypertensive medications, the advantage being that these drugs modulate the renin-angiotensin-aldosterone axis and are considered cardioprotective [3]. In addition, ACEIs/ARBs are also used to treat patients with chronic left ventricular dysfunction and those with diabetic nephropathy. However, blunting of the renin-angiotensin-aldosterone axis may cause hypotension during induction of anaesthesia [3].

Continuation of ACEIs/ARBS in the perioperative period is associated with intraoperative and postoperative hypotension, and thus, many clinicians choose to withhold administration of these drugs [4]. However, this is often based on individual practice and has no relation to the profile of the patient and/or the surgery performed [3]. In addition, there is variation in practice with regard to the time of discontinuation with some clinicians discontinuing the drugs 24 h before surgery while some others choose to skip a morning dose of the drug. This in turn is not related to the pharmacokinetic or pharmacodynamic properties of the drugs. Other evidence points to the beneficial effects of a continuation of these drugs in the perioperative period with the demonstration of improved outcomes [5]. While perioperative hypotension is certainly a concern with the continuation of ACEIs/ARBs, discontinuation of these drugs before surgery makes patients susceptible to postoperative hypertension when the effects of anaesthetics wear off, and this may cause harm by increasing the risk of myocardial ischaemia [6]. In fact, postoperative hypertension is more frequently seen in those with preoperative hypertension and is known to increase the risk of occurrence of major adverse cardiovascular events [7].

This trial is thus planned to study the effect of continuing vs withholding the ACEI/ARBs on mortality and other major outcomes in hypertensive patients undergoing elective non-cardiac surgery.

## Study hypothesis

A previous retrospective study had found that withholding ACEIs/ARBs 24 h before non-cardiac surgery is associated with a reduction in all-cause mortality [3]. With this background, the primary hypothesis is that discontinuation of ACEIs/ARBs > 24 h before surgery will result in a lower all-cause 30-day mortality and major adverse cardiovascular and non-cardiovascular events. The secondary hypothesis is that there will be a decreased incidence of adverse events like intraoperative hypotension, angioneurotic oedema, and myocardial injury in patients in whom the ACEIs/ARBs are discontinued, and there would be shorter ICU and hospital stay duration. The impact of withholding of ACEIs/ARBs on case cancellations due to rebound hypertension will also be evaluated.

## Objectives

The primary objective of this study is to evaluate the effect of continuing vs withholding of ACEIs/ARBs on the composite all-cause mortality, major adverse cardiovascular outcomes (acute myocardial infarction, stroke, arrhythmias requiring intervention, hypertensive crises, acute pulmonary oedema, postoperative cardiogenic shock), acute kidney injury, postoperative respiratory failure, sepsis and unplanned ICU admissions in hypertensive patients undergoing major non-cardiac surgery. The secondary objective will be to evaluate the incidence of intraoperative hypotension, myocardial injury, angioneurotic oedema, case cancellations and duration of ICU and hospital stay.

## Methods

### Study design

**Fig. 1:**
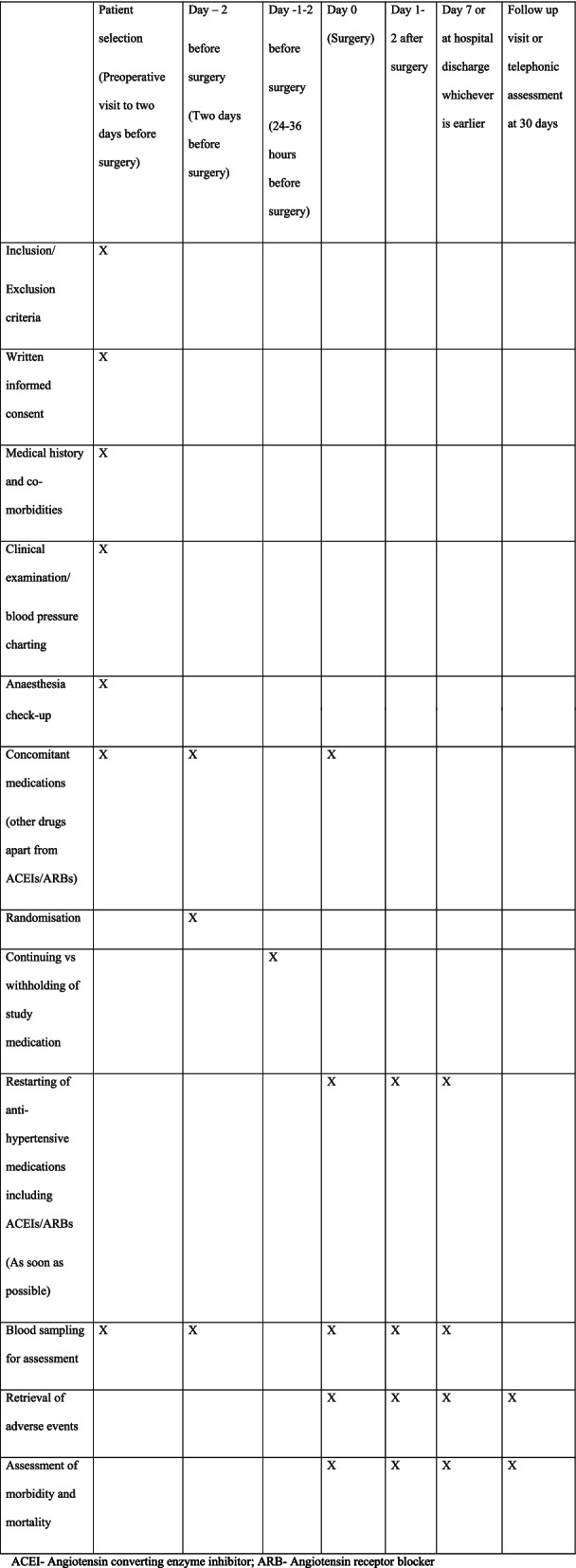
SPIRIT figure

Continuing vs withholding ACEIs/ARBs is a multi-centric, open-label, randomised controlled two-arm trial, with a 1:1 allocation of hypertensive patients undergoing major non-cardiac surgery to either continue or stop their ACEIs/ARBs. When admitted to the hospital, patients will be randomised to either continue their ACEIs/ARBs including on the day of surgery (group A) or discontinue the drugs 24–36 h before surgery (group B). The primary endpoint will be a composite outcome of all-cause 30-day mortality, major adverse cardiovascular events (acute myocardial infarction, stroke, arrhythmias requiring intervention, hypertensive crises, acute pulmonary oedema, postoperative cardiogenic shock), acute kidney injury, postoperative respiratory failure, sepsis and unplanned ICU admissions. The Standard Protocol Items: Recommendations for Interventional Trials (SPIRIT) checklist is provided in Additional file 2, and the schedule of the participant enrolment in the study (SPIRIT figure) is shown in Fig. 1.

### Settings

The trial will be conducted in three tertiary care hospitals in India: All India Institute of Medical Sciences Bhubaneswar, JIPMER Puducherry and CMC Vellore. All three centres are high-volume centres and have dedicated anaesthesiologists caring routinely for patients with various co-morbidities undergoing non-cardiac surgery.

### Participants

**Fig. 2:**
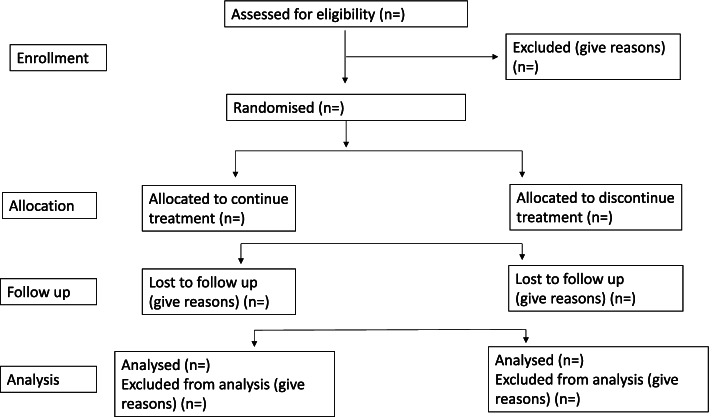
Enrolled adult hypertensive patients undergoing elective major non-cardiac surgery under general or neuraxial anaesthesia over a period of 3 years

We aim to enrol 2100 adult hypertensive patients undergoing elective major non-cardiac surgery under general or neuraxial anaesthesia over a period of 3 years (Fig. 2). Eligible patients will be screened and enrolled during their preoperative visit, and the project staff will follow up with the patient before surgery to ensure randomisation and compliance with the group allocation. Patients who are allocated to group A (continuing the ACEIs/ARBs) will continue to take these medications as per schedule including the day of surgery. For those who are allocated to group B (discontinuing the ACEIs/ARBs), these drugs will be omitted 24–36 h prior to surgery. Patients will then be followed up in-hospital by the investigators and at 30 days by telephonic calls by the project staff.

### Inclusion criteria

The following are the inclusion criteria:

1. Patients requiring major elective non-cardiac surgery are defined as surgery with an expected duration of > 2 h from the surgical incision and/or a postoperative hospital stay of at least 3 days [8].
2. Age 18–70 years of either gender.
3. Patients chronically treated with ACEIs/ARBs (ideally 3 months or more or a minimum of 2 weeks at the time of randomisation).

### Exclusion criteria

The following are the exclusion criteria:

1. Emergency surgery (surgical treatment needed within 48 h)
2. Hyperkalaemia (serum potassium level > 5.5 mmol/L) during the preoperative visit
3. Patients with severe chronic renal insufficiency as defined by estimated glomerular filtration rate < 15 mL/min/1.73 m^2^ or requiring renal replacement therapy
4. Patients with preoperative shock (defined by the need for vasoactive drugs before surgery or a mean arterial pressure < 65 mmHg)
5. Patients taking ACEI/ARBs for left ventricular dysfunction (ejection fraction < 50%)
6. Uncontrolled preoperative blood pressures (systolic blood pressure > 180 mmHg and/or diastolic blood pressures of > 110 mmHg)

### Ethical aspects

The study was approved by the Institutional Ethics Committee of AIIMS Bhubaneswar (1 June 2020 vide letter no. T/EMF/Anesth/20/10) and by the ethics committee of each of the collaborating hospitals. Furthermore, the study was registered in the Clinical Trials Registry of India (CTRI/2021/01/030199, registered on 4 January 2021). Patients will receive written information regarding the trial, the possible risks and benefits, and an informed consent will be obtained from them by the research assistants before enrolling them into the trial.

### Randomisation and blinding

All study patients will receive the same standards of care and management, including treatment of any harm resulting from the trial. However, they will receive no compensation as a result of participation in the study. They will be randomised to either continue with their ACEI/ARB treatment (including on the day of surgery) (group A) or to withhold it 24–36 h before surgery (group B) (two doses of the ACEI or ARB will be omitted to account for variation in the timing of administration, i.e. morning or evening). The patient’s blood pressure will be monitored periodically and treated with calcium channel blockers if necessary. Other antihypertensives that the patient may be concurrently receiving will be continued on the day of surgery. All such orders will also be recorded. Following surgery, anti-hypertensive medications including ACEIs/ARBs will be restarted at the earliest and the date of restarting anti-hypertensive therapy will be noted.

Randomisation will be carried out using permuted block randomisation (blocks of 20). The randomisation codes and sequence will be computer-generated by a statistician at each institute. The randomisation codes and the sequence will be kept secure at each participating centre. One code from a block sequence will be allotted each time when the patient is enrolled into the study. Allocation concealment will be with opaque sealed envelopes which will be opened 2 days before surgery. The diagnosis of outcomes will be made by clinicians treating the patients. The data for primary and secondary outcomes will be collected from the anaesthesia and surgical case records by the investigators and the project staff. The project staff will be trained by the investigators in the aspects of data collection and approach to missing data.

### Data collection and follow-up

All information required according to the protocol will be entered in the paper-based case report forms. The data will be collected as and when they are obtained and recorded in these case report forms. Data entry will be performed on electronic media. Each missing data item will be coded. Clinical research assistants will carry out regular follow-up visits at the study sites. The approach to missing data will be aggressive, and the investigators will attempt to recover/retrieve any missing data by contacting all the patients or their next-of-kin in case of discharge from the hospital. In case of withdrawal of consent, the data of the patient will not be analysed. Data of each patient will be anonymised and use the initials of the patient and the unique code assigned to him or her. All serious adverse reactions (e.g. angioedema) will be brought to the notice of the ethics committees of the respective institutions, as well as the Data Safety and Monitoring Board (DSMB).

### Clinical data

Baseline data such as age, gender, height, weight, BMI, ASA physical status, surgery, co-morbidities and anti-hypertensive medications will be collected. In-hospital mortality/mortality within 30 days of surgery, postoperative major adverse cardiovascular events (acute myocardial infarction, stroke, acute pulmonary oedema, postoperative cardiogenic shock, acute severe hypertension crisis, severe cardiac arrhythmia requiring therapeutic intervention), the incidence of intra- and postoperative (24 h) hypotension (systolic blood pressure < 90 mmHg), acute kidney injury (based on the serum creatinine item of the KDIGO criteria [9]; baseline serum creatinine will be the preoperative value), hyperkalaemia (serum potassium level > 5.5 meq/L requiring intervention), angioneurotic oedema, sepsis (suspected or culture-positive), postoperative respiratory failure and unplanned ICU admissions will be recorded.

The impact of withholding of ACEIs/ARBs on rebound pre-induction hypertension (blood pressure > 160/90 mmHg) on the day of surgery and case cancellation will also be evaluated. Intraoperatively, the episodes of hypotension will be treated with vasopressors (ephedrine, phenylephrine, norepinephrine and epinephrine). The choice of vasopressor will be left at the discretion of the anaesthesiologist. The lowest arterial pressure, duration of hypotension and total doses of vasopressors (bolus and infusion) will also be collected and reported. Other secondary outcomes noted will be myocardial injury (elevation of serum troponin-t evaluated till 12/24 h after surgery), duration of primary hospital stay and duration of ICU stay. The ancillary and post-trial care of the participants will be as per the standard of care for all patients admitted to the hospital for routine non-cardiac surgery, till their duration of stay in the hospital.

### Efficacy end-points

The composite primary outcome and the secondary outcomes will be collected for each patient. Diagnosis of complications will be made by the treating physicians. A data adjudication committee will review the appropriateness of the collected data for each outcome measure. Members of the committee will be blinded to the group allocation and comprise non-participating clinicians from each centre.

### Strategies to ensure adequate enrolment and protocol compliance

Case record forms will be uploaded into a secure database at the trial coordination centre in a time-bound manner. This will allow regular checks for enrolment rates, data accuracy and protocol adherence. Each centre will need to recruit approximately 700 patients. The study duration will be for a period of 3 years, extendable by 1 year in case the sample size is not met. We will perform an interim analysis after 1000 patients to assess the futility or harm of the trial. The O’Brien/Fleming method for interim analysis will be used [10].

### Auditing trial conduct

Auditing of the trial at each site will be carried out at 6-monthly intervals. The trial auditors will be independent of the investigators and comprise representatives from the Institutional Review Board. Trial auditing will be done with respect to accuracy and completeness of case record forms, verification of informed consent process and documentation, adverse event reporting and protocol adherence.

### Data safety and monitoring board

DSMB meetings will be held twice or thrice a year as per the institutional policy, and any occurrence of adverse events during the study will be discussed and necessary action will be taken. In general, the following aspects will be addressed by the DSMB.

1. Monitoring safe and effective conduct of treatments of the study participants
2. Analysis of adverse events
3. If necessary, recommending early conclusion of a trial when significant benefits or risks have been demonstrated
4. Blinded trial interim result analysis

### Statistical analysis

To adjust for confounders, a multivariate logistic regression analysis will be carried out, stratifying for ASA physical status, age, gender, general vs neuraxial anaesthesia and types of surgery. Data will be also stratified according to the centre. Attempts to account for missing data will be made, but since this will be an intention to treat analysis, all data that will be available from each patient will be included in the study.

### Sample size calculation

Based on an incidence of 25% of the primary endpoint in the reference group [11], a total of 2016 patients will allow an 80% power to detect a 21% relative decrease of complications in the experimental group. To account for dropouts, a total of 2100 patients (700 at each centre) will be included. The analysis will be an intention-to-treat analysis. Since one interim analysis will be planned after recruiting 1000 patients according to the O’Brien and Fleming method, the interim alpha will be set at 0.0052 and the final nominal alpha at 0.048 (Additional file 1). The *P* values and respective sample sizes were based on a final sample size of 2100, and 1 interim analysis at halfway point was calculated using the “gsDesign” package of the R statistical software v4.0.0 (R Statistical Corp, Vienna, Austria).

### Protocol amendments

Any protocol amendments (outcomes, eligibility criteria, analyses) subsequent to starting the trial will be communicated to each participating centre’s ethics committee, and after obtaining the necessary approval, the same will be amended in the trial registry.

### Strengths and limitations of the study protocol and dissemination of data

The advantage of this trial will be the large sample size and the multi-centric nature leading to wider generalisability. The results of this trial will be sent for publication in peer-reviewed journals. All investigators satisfying the authorship criteria as per the ICMJE criteria will be credited with authorship, while others who do not meet the criteria, but have helped in the study, will be acknowledged either individually or as a group. Access to the participant dataset will be shared with journals on request.

## Discussion

In 2017, the results of the VISION trial showed that withholding the drug during the 24-h preoperative period was associated with a reduction in 30-day mortality and major cardiovascular events as compared to those in whom the drug was continued, although the incidence of perioperative hypotension was similar between the two groups [3]. However, the primary trial was a prospective trial of vascular events in patients undergoing non-cardiac surgery, and the findings of continuing vs withholding of ACEIs/ARBs were reported as sub-group analysis. Subsequent systematic reviews have not been able to show a difference in the mortality and other adverse outcomes in patients in whom these drugs were withheld vs those in whom they were continued [12].

There is considerable variability in the recommendations in the guidelines of various societies with regard to continuing vs withholding of ACEIs/ARBs in surgical patients. While the 2014 American College of Cardiology/American Heart Association guidelines suggest that it is reasonable to continue therapy preoperatively, and re-institution of therapy early in the postoperative period [13], the recent Canadian Cardiovascular Society guidelines state that these drugs should be omitted 24 h before surgery [14]. In contrast, the European Society of Cardiology/European Society of Anaesthesiology bases its recommendations on the indication for treatment with an ACEI/ARB, recommending discontinuation for 24 h before surgery if prescribed for hypertension and continuation if prescribed for heart failure and left ventricular systolic dysfunction [15]. Unfortunately, the evidence for the American, European, and Canadian guidelines is based largely on retrospective studies with a strong risk of bias, small randomised trials with a limited number of patients or observational studies and, thus, limited in nature.

To our knowledge, only two multicentric trials (ongoing) are looking at the effect of continuing vs withholding of ACEIs/ARBs in patients undergoing non-cardiac surgery [11, 16]. However, the protocol of our study differs from the other two protocols. In the STOP-or-NOT trial encompassing 30 French centres, the ACEI/ARBs will be withheld 48 h before non-cardiac surgery [11]. In contrast, our trial will evaluate the continuing vs withholding of ACEI/ARBs 24–36 h before non-cardiac surgery, similar to the retrospective VISION trial which also evaluated the withholding of the ACEIs/ARBs 24 h before surgery on major adverse outcomes [3]. The second ongoing multicentric study is published by Yang et al. encompassing six Chinese centres which includes geriatric hypertensive patients (60–80 years), and their primary end-point is the incidence of perioperative hypotensive events in patients undergoing a general anaesthetic [16]; whereas our primary outcome is a composite outcome of all-cause in-hospital/30-day mortality, major adverse cardiovascular and non-cardiovascular events in patients aged 18–70 years and undergoing surgery under both general and neuraxial anaesthesia. Thus, the result of our study is expected to add value to this important clinical conundrum.

## Trial status

Protocol version and date: version 2 dated 02-07-2022

Date recruitment began: not yet recruiting

Estimated date of completion of recruitment: 31-12-2025

## Supplementary Information

**Additional file 1. **The *P* values and respective sample sizes based on a final sample size of 2100, and 1 interim analysis at halfway point was calculated using the “gsDesign” package of the R statistical software v4.0.0 (R Statistical Corp, Vienna, Austria).

**Additional file 2.** SPIRIT 2013 Checklist: Recommended items to address in a clinical trial protocol and related documents*.

## Data Availability

Datasets are not yet generated for this planned study.
