# Supplementary material for: The effect of continuing versus withholding angiotensin-converting enzyme inhibitors/angiotensin II receptor blockers on mortality and major adverse cardiovascular events in hypertensive patients undergoing elective non-cardiac surgery: study protocol for a multi-centric open-label randomised controlled trial
Source: Trials. 2022 Aug 17;23:670. doi: 10.1186/s13063-022-06616-y (PMC9386985; doi:10.1186/s13063-022-06616-y)
Supplement: Supplementary file 1 — Additional file 1. The P values and respective sample sizes based on a final sample size of 2100, and 1 interim analysis at halfway point was calculated using the “gsDesign” package of the R statistical software v4.0.0 (R Statistical Corp, Vienna, Austria). [file 13063_2022_6616_MOESM1_ESM.docx]

The P values and respective sample sizes based on a final sample size of 2100, and 1 interim analysis at halfway point was calculated using the "gsDesign" package of the R statistical software v4.0.0 (R Statistical Corp, Vienna, Austria).

Number of analyses (Including final) = 2

Test-Type = 2 tailed

Method = "O'Brien and Fleming"

Beta = 0.2

Fixed sample size = 2100

Result: Symmetric two-sided group sequential design with 80 % power and 2.5 % Type I Error. Spending computations assume trial stops if a bound is crossed.

| Analysis | N | Z | P | Spend |
| --- | --- | --- | --- | --- |
| 1 | 1059 | 2.80 | 0.0026 | 0.0026 |
| 2 | 2118 | 1.98 | 0.024 | 0.0224 |
| Total |  |  |  | 0.025 |

P value (Interim analysis): 0.0052; P value (Final analysis): 0.048

Thus, the final sample size and the p values at which data will be considered significant are 1059 (p <0.0052) and 2118 (p<0.048) respectively. If the P value crosses the boundary at any of the analyses, the trial will be stopped and be deemed significant.
